# The role of the radiologist in diagnosing placenta percreta: a case report and review of the literature

**DOI:** 10.1093/jscr/rjad319

**Published:** 2023-06-07

**Authors:** Yahya Elharras, Safaa Choayb, Najlae Lrhorfi, Nazik Allali, Latifa Chat, Siham Elhaddad

**Affiliations:** Pediatric Radiology Department, Children’s Hospital, University Mohammed V, Rabat, Morroco; Pediatric Radiology Department, Children’s Hospital, University Mohammed V, Rabat, Morroco; Pediatric Radiology Department, Children’s Hospital, University Mohammed V, Rabat, Morroco; Pediatric Radiology Department, Children’s Hospital, University Mohammed V, Rabat, Morroco; Pediatric Radiology Department, Children’s Hospital, University Mohammed V, Rabat, Morroco; Pediatric Radiology Department, Children’s Hospital, University Mohammed V, Rabat, Morroco

**Keywords:** placenta previa, percreta, abnormal placental insertion, ultrasound, MRI

## Abstract

Placenta percreta is the most severe and least common form of placental insertion abnormalities. The increasing frequency of C-Section deliveries has led to more of these abnormalities. Ultrasound and magnetic resonance imaging (MRI) have a key role in diagnosing these abnormal adherences since it shows best transmural extension of the placental tissue. We report a case of a woman with a previous cesarean delivery who had been diagnosed with a placenta preavia on ultrasound and a suspicion of transmural extension with her MRI later showing a placenta percreta.

## INTRODUCTION

Due to the increasing number of cesarean deliveries, abnormal placental insertion has also increased in frequency becoming one common cause of postpartum hemorrhage and peripartum morbidity. It includes three grades based on the severity of placental invasion: increta, accreta and percreta. 

The latter is a term given to the most severe but least common form of the spectrum of abnormal placental villous adherence, where there is a transmural extension of placental tissue across the myometrium with a serosal breach. This extension can only be seen on ultrasound and magnetic resonance imaging (MRI) that highlights the role of the radiologist in the diagnosis.

## CASE REPORT

**Figure 1:**
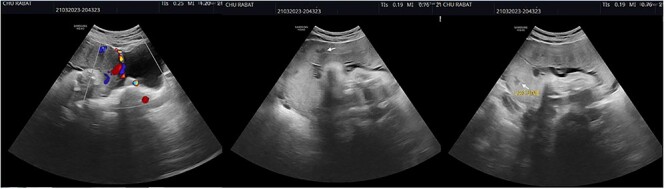
Ultrasound showing intra-placental lacunae (white arrows) as well as placental tissue protrusion beyond the uterine myometrium, as well as urinary bladder wall discontinuity and the extension of Doppler vascularity between serosa and the bladder (Fig. 1).

We report the case of a 30-year-old woman who had a C-Section 2 years earlier for fetal macrosomia. She presented in our department for a normal routine fetal ultrasound that identified some intra-placental lacunae as well as the protrusion of placental tissue beyond the uterine myometrium, with a discontinuity in the urinary bladder wall and the extension of Doppler vascularity between serosa and the bladder (Fig. 1). Abnormal placental insertion was thus suspected and a placental MRI was realized to confirm the diagnosis.

**Figure 2:**
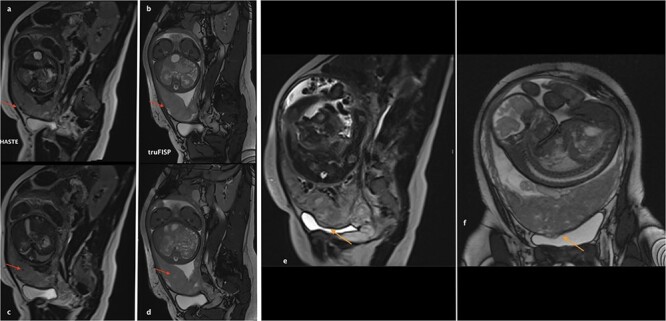
(**a**)–(**d**) Sagittal sequences showing dark intra-placental bands (arrows) in low signal in HASTE sequences and in high signal on truFISP. (**e**) Sagittal HASTE and (**f**) coronal truFISP sequences showing myometrial thickening and discontinuity in the bladder wall as well as its invasion by the placental tissue (arrow).

Her MRI showed some thick linear and round bands arising from the maternal side of the placenta seen in low signal in single-shot fast spin echo (SSFSE) T2-Weighted images (HASTE) and in high signal on Balanced Steady State Free Precession (bSSFP) (True Fisp), corresponding to dark intra-placental bands (Fig. 2). The most important finding was myometrial thinning and the bulging of the placenta within the Cesarean scar as well discontinuity in the urinary bladder wall with its invasion by the placental tissue (Fig. 2). These abnormalities were associated to some tortuous bands in a signal on all sequences corresponding to vessels. The diagnosis of placenta percreta was then made and the patient was referred to the obstetrics department for urgent care.

## DISCUSSION

**Figure 3:**
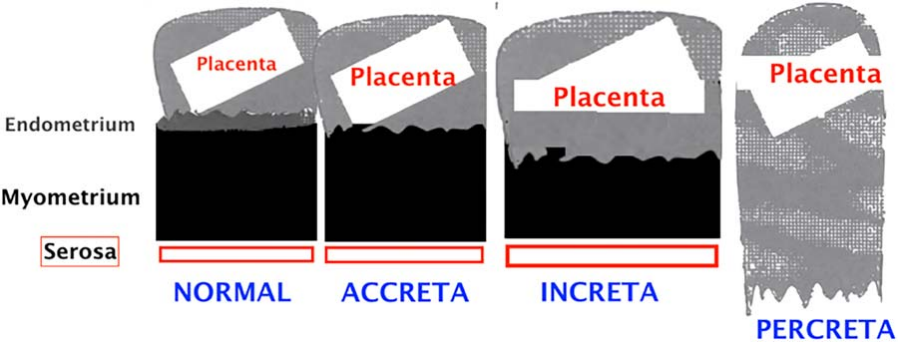
Schematic representation of the different types of placental insertion abnormalities.

Placenta percreta is the most severe, yet least common, form of abnormal placental insertion (or placental villi adherence abnormalities) (Fig. 3). It is defined as an extension of placental tissue, by a breach in the serosa, across the myometrium, and it represents the main cause of peripartum morbidity. Due to the rising of C-section delivery rate, the incidence of placenta percreta is also increasing. Other risk factors include placenta praevia, advanced maternal age, previous pelvic surgery that may cause intra uterine adhesion bands.

Histopathological examination remains the main exam to confirm the diagnosis, but unfortunately, this can only be confirmed in the event of hysterectomy, while nowadays conservative treatment is favored [1].

Normal placenta echo structure changes during pregnancy. In the second trimester, it is homogenously hyperechoic while in the third trimester, it turns to a more heterogeneous calcified aspect associated to physiological placental lakes (round with hyperechoic wall appearance without Doppler vascularization) [2]. On the other hand, the myometrium is normally hypoechoic and there’s a space between it and the placental bed called the retroplacental clear space, which is continuous and more hypoechoic compared to the myometrium.

In the case of placenta percreta, ultrasound may show the loss of retroplacental clear space, a reduced myometrial thickness as well as placental lacunae. The latter finding corresponds to parallel linear vascular channels extending from the placental bed into the myometrium. These structures tend to show turbulent flow in Doppler and ‘Swiss cheese’ aspect (if there are many of them) [3]. These lacunae should not be confused with physiological vascular lakes. Other ultrasound findings that may orientate are an increased vascularity between serosa and adjacent structures essentially the bladder as well as placental tissue protrusion beyond the outer confines of the myometrium [4].

Ultrasound in diagnosing placental percreta by ultrasound has its limitations, which has led to introducing the MRI as complementary imaging tool. Thorp *et al*. published in 1992 the first case of MRI diagnosis of placenta percreta on a former placenta previa [5].

T2-WI are taken in the three planes: strict sagittal, axial and coronal. Placental invasion is best seen using T2-weighted sequences without fat suppression. bSSFP sequences (named True Fisp on Siemens and Fast Imaging Employing Steady-state Acquisition (FIESTA) on General Electric (GE)) and fast sequences (such as SSFSE on GE and Haste on Siemens) have a huge advantage of being very little artefacted by maternal and fetal movements [1].

**Figure 4:**
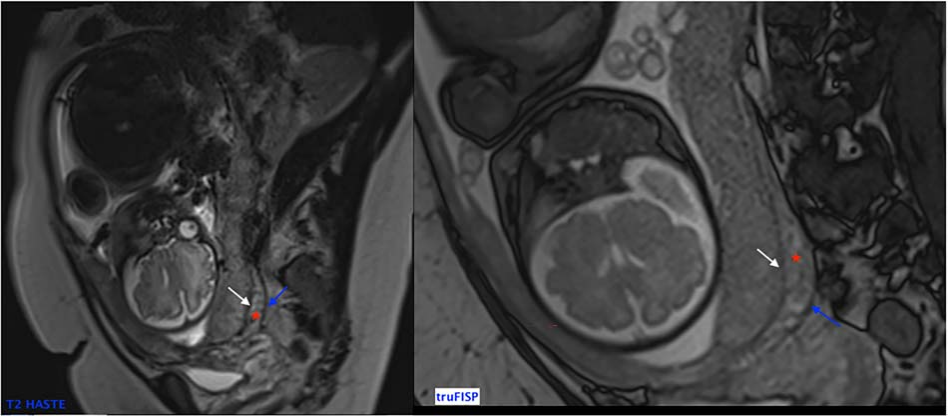
Sagittal T2 Weighted Images showing normal MRI signal of the placenta and the myometrium at the end of 2nd trimester. Note the trilaminar aspect of the myometrium: retro placental layer in hyposignal (white arrow), intermediate layer (the zone of arcuate vessels in intermediate signal red asterisk) and the external layer (the serosa) with a thin T2 hyposignal aspect (blue arrow).

Diffusion weighted images can also be used to better see contours of the placenta [6]. It is important to note that MRI must be performed early enough to be able to plan childbirth and in order to decrease the false-positives (physiological placental changes at the end of pregnancy). Alamo *et al*. suggested performing it before 35 weeks of pregnancy while Lim *et al*. proposed before 30 weeks [7, 8]. Normal MRI signal of the placenta during at the end of 2nd trimester is a homogeneous isosignal aspect respecting the myometrium on T2-WI. As for the 3rd trimester, the placenta begins to have a more T2 hyposignal lobular and heterogeneous aspect. The myometrium usually has high signal on T2-WI (Fig. 4) with a trilaminar appearance.

**Figure 5:**
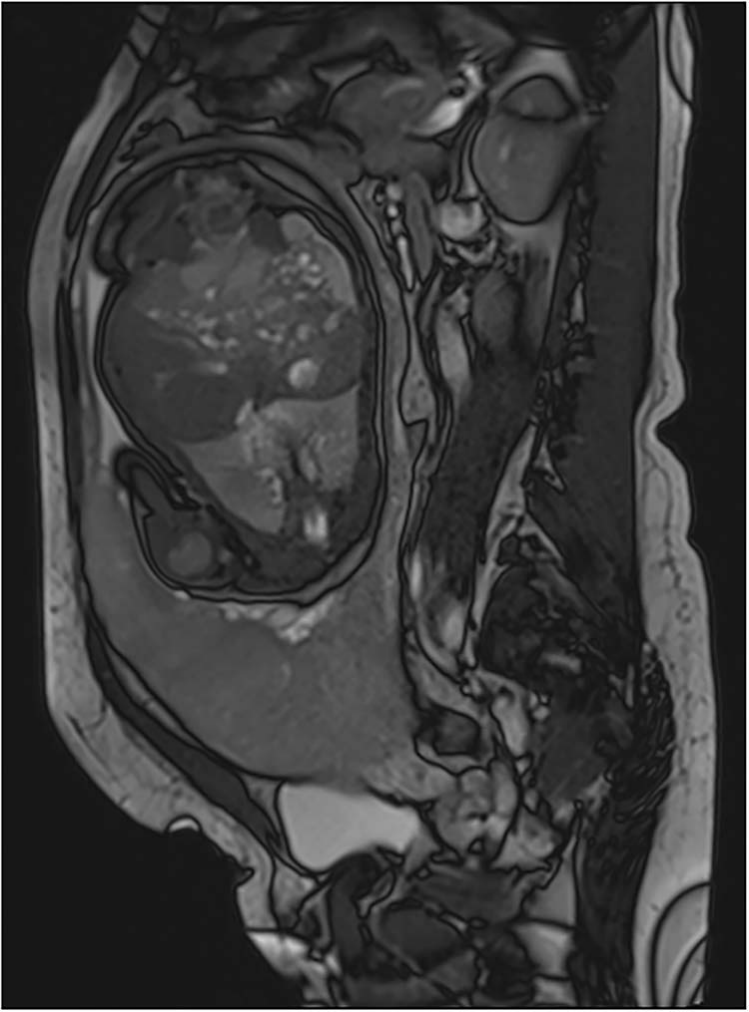
Trufisp T2 sagittal-WI of a 44-year-old patient in our department showing the invasion of the urinary bladder.

In the case of placenta percreta (like ultrasound), we might have dark intra-placental bands on T2-WI with a heterogenous appearance of the placenta and abnormal disorganized placental vascularity seen as traversing vessels. There’s also focal interruptions of the myometrial wall and the most particular sign in placenta percreta is the tenting of urinary bladder with the involvement of uterine serosa or extra-uterine involvement of adjacent organs like bladder, rectum or abdominal wall [9] (Fig. 5).

## CONCLUSION

This uncommon pathology requires a precise diagnosis in order to guide the right obstetrical care. Surgical intervention is the pillar of treatment in most cases, but bleeding during the intervention is a serious concern particularly in situations where the bladder or (other adjacent structures) are involved. Imaging remains key in diagnosing placenta percreta with ultrasound being the first-line examination and MRI coming in second when the diagnosis is uncertain or when the myometrial wall extension assessment is incomplete on ultrasound.

## ACKNOWLEDGMENTS AND AUTHORS' CONTRIBUTIONS

El Harras Yahya was responsible for manuscript concept, design as well as editing and literature search. Safaa Choayb helped in manuscript editing and literature search, manuscript editing and manuscript review. Najlae Lrhorfi contributed to conception and design. Nazik Allali contributed to acquisition, analysis and interpretation. Latifa Chat critically revised the manuscript and gave final approval. Siham El Haddad contributed to acquisition, analysis and interpretation; critically revised the manuscript and gave final approval.

## CONFLICT OF INTEREST STATEMENT

The authors declare that they have no known competing financial interests or personal relationships that could have appeared to influence the work reported in this paper.

## FUNDING

This research received no specific grant from any funding agency in the public, commercial or not-for-profit sectors.
